# The Recent Meeting of the Texas State Medical Association

**Published:** 1890-05

**Authors:** 


F. E. DANIEL, M. D., EDITOR AND PUBLISHER.

THIS JOURNAL, by unanimous vote of the members, was made the Official Organ of the Austin District Medical Society.

COLLABORATORS:

*Dr. R. M. Swearingen, Austin.*
*Prof. B. E. Hadra, M. D., Galveston.*
*Prof. Geo. Cupples, M. D., San Antonio.*
*Dr. T. C. Osborn, Cleburne, Texas.*
*Dr E. J. Doering, Chicago.*
*Dr. E. J. Beall, Fort Worth.*
*Dr Odo Betz, Germany.*
*Dr. J. W. McLaughlin, Austin.*
*Dr. T. J. Tyner, Austin.*
*Prof. J. F. Y. Paine, M. D., Galveston.*
*Dr. R. H. L. Bibb, Mexico.*
*Dr. T. J. Bennett. Austin.*
*Dr. Bat Smith, Wharton.*
*Dr. E. Meierhof, New York.*
*Prof. W. B. Rogers, M. D., Memphis, Tenn.*

### THE RECENT MEETING OF THE TEXAS STATE MEDICAL ASSOCIATION.

The twenty-second annnal meeting of the State Medical Association held at Fort Worth the last week in April, was one of the most successful, scientifically, that has been held in several years. No hitch or halt occurred, and all work went as smoothly as possible. Perhaps a larger number of papers of real value were read than at the average meeting; and the plan of having the whole time given up to section work except the morning of the first day, devoted to reports of officers and committees, and an executive session the last day, proved to be eminently satisfactory.

A synopsis of the proceedings is published in this issue. The citizens of Fort Worth, and especially her medical men deserve the grateful thanks of the Association for the cordial reception given them, and for the very enjoyable social entertainment provided for members and their ladies. The banquet was superb, and in every way worthy of this hospitable and warm-hearted people. They could not help its raining, its true; and they managed to make everybody comfortable and happy, despite the torrents of rain that fell first, last and all the time we were there. We dare say the most captious and fault finding member could not "pick a flaw" in the management of the reception and entertainment. All left well pleased, and with a high opinion of the city, its people, and especially of the reception committee.

The exhibition of surgical instruments and pharmaceutical preparations was good, though not as large as on some former occasions. We missed Parke, Davis & Co., W. R. Warner & Co., Reed & Carnrick, Doliber & Goodale Co., and some others; and they missed a "good thing," perhaps; for there were present, first and last, nearly or quite five hundred physicians. But the old reliable—Sharpe & Dohme was on hand as usual; also Tarrant & Co., "Maltine," "Lactopeptine," and many others, with their courteous representatives, and a splendid exhibit. If we omit to mention any of our friends who had an exhibit there, they must pardon us, for the display was across the street, in the Natatorium building, and besides the writer's being kept busy every minute, it rained so that he could not get an opportunity to visit the hall at all. The Transactions will be a larger and better volume than usual, and the publishing committee will endeavor to get it out earlier than heretofore; say by 1st of August.

We have neither space nor inclination to discuss anything that was done on the occasion; a perusal of the minutes published herewith, will give the facts; but we cannot help congratulating the Association on the eminently sensible resolution which was adopted authorizing the secretary to employ a stenographer at each meeting to secure the discussions on papers read. This will add greatly to the interest and value of the published Transactions; and any reasonable man may see how impossible it is for the Secretary, unless he had the eyes of Argus and the many hands of Briarius, occupied as he is, every moment of time, to take down the exact and full remarks of members.

Already we look forward to the Waco meeting next April, with pleasing anticipation.

